# Audit of Motor Vehicle Accidents at a Trauma Center

**DOI:** 10.7759/cureus.23729

**Published:** 2022-04-01

**Authors:** Shaurav Ghosh, Farah Naaz Kazi, J V Pranav Sharma

**Affiliations:** 1 General Surgery, Vydehi Institute of Medical Sciences and Research Centre, Bangalore, IND; 2 Surgery, Vydehi Institute of Medical Sciences and Research Centre, Bangalore, IND

**Keywords:** in-hospital length of stay, mortality, injury severity score, obesity, fall

## Abstract

Background

Motor vehicle accidents (MVAs) are the leading cause of accidental deaths in India. An audit of trauma cases is required in order to improve hospital policy and patient care, as well as to change the attitude and perspective of healthcare staff.

Methods

A retrospective observational study was performed on MVA trauma victims admitted to a tertiary trauma care center. Parameters included mean age with range, gender distribution, length of hospital stay (LOS), anatomical location of injuries, and the percentage of age groups and number of patients undergoing a nonoperative approach versus those with exploration and intervention. The correlation of body mass index (BMI) and co-morbidities with trauma, whole-body CT scan (WBCT) versus selective scanning, readmissions and revisits, and blood transfusion requirements were studied.

Results

The majority of patients were young male adults and females having a higher LOS than males. Prolonged hospitalization is linked to a higher risk of complications and a higher expense. Individuals who suffered severe injuries recovered more slowly 12 months after the accident. The majority of patients had a brief hospitalization. Sixty-two point three (62.3) percent of patients suffered a head injury, with men accounting for the majority. Men were worst-affected, necessitating surgery. Obesity and BMI, regardless of gender, are not associated with trauma outcomes. Our studies found no link between co-morbidities and length of stay in MVA patients. Although the majority of patients did not require surgery, 28.8% required a blood transfusion. Our research found no link between BMI and injury severity score (ISS).

Conclusion

Obese people sustaining MVAs had the same injured body regions as patients who were normal weight. They had a lower ISS than normal-weight individuals but a lengthier in-hospital stay.

## Introduction

The Groningen Trauma Study group showed that during a 24-year period, the largest incidence rate of trauma was among men aged 20-29 years [1]. The male-to-female ratio was 1.8, and the mortality rate was 0.5%, with women over 70 years old having the greatest mortality rate. Chassé M et al. showed that higher BMI in women may be a risk factor [2]. Ntundu SH et al. further showed that blunt abdominal injury was commonly linked to road traffic accidents (RTAs) [3-10]. Mortality was linked to extra-abdominal injury, injury to the head or pelvis, length of stay (LOS) ≥ seven days, systolic BP < 90, and anemia. A delay in admission of > six hours strongly predicted mortality. Chen AK et al. found no difference in the severity of solid organ injury following blunt abdominal trauma between obese and non-obese patients [3]. Obese individuals had a higher rate of procedural intervention and mortality. According to Ramakrishnan VT et al., early detection of coagulopathy and management with prompt bleeding control increases the survival of trauma patients [11-12]. Chuang et al. showed that injured obese patients had similar wounded body regions as normal-weight patients [4]. However, they had a lower Injury severity score and longer hospital stay. According to Davie et al., the rate of recovery in 24 months following injury was identical for those with no or one pre-existing co-morbidity but much slower for those with multi-morbidity [5-6].

In the above studies, the trauma registry was utilized to collect data for ongoing trauma care research that could be used for quality control and planning. According to the National Crime Records Bureau (India), the fatalities in road accidents have increased by 1.3% in 2019 [7]. However, the lack of a high-quality database has hampered the development and implementation of hospital policies and patient care administration. In order to improve the quality of care, we choose to investigate the epidemiology of in-patients who had been involved in motor vehicle accidents by reviewing motor vehicle accident (MVA) data from the general surgery trauma registry.

## Materials and methods

Aim, design, and setting

This was an epidemiological study of motor vehicle accidents in patients at a tertiary trauma center. It was a retrospective clinical study conducted at a teaching hospital and tertiary care referral trauma center.

Methodology

Data were collected retrospectively using the trauma registry of the Department of General Surgery of Vydehi Institute of Medical Sciences and Research Centre, Bangalore, following an MVA between 2018 and 2019.

Inclusion Criteria

All patients met with a motor car accident between 2018 and 2019, including all kinds of bodily trauma.

Exclusion Criteria

This study excluded outpatient department (OPD) and comatose cases. The most crucial step in this study has been to rule out the exact factors causing the accident based on the next of kin information.

Statistical methods

Descriptive and inferential statistical analysis was done. Results on continuous measurements are presented on mean ± SD (Min-Max) and categorical measurements are presented in Number (%). Significance is assessed at a 5% level of significance. The student's t-test (two-tailed, independent) and chi-square/Fisher's exact test were used for data analysis. Fisher's exact test is used when cell samples are very small. Suggestive significance for P-value 0.05<P<0.10, moderately significant for P-value 0.01<P £ 0.05, and strongly significant for P-value P£0.01.

## Results

Table 1 shows the age distribution in our study. Patients less than 30 years proved to have more fatality considering they drive at a faster speed.

**Table 1:** Age distribution of patients studied Mean ± SD: 35.88±15.91

Age in Years	No. of Patients	%
<30	22	41.5
30-40	15	28.3
41-50	7	13.2
>50	9	17.0
Total	53	100.0

Table 2 projects that 69.8% of the patients who had a motor car accident were male as compared to a mere 30% of females. This pattern has been observed in a lot of studies [10-14].

**Table 2:** Gender distribution of patients studied

Gender	No. of Patients	%
Female	16	30.2
Male	37	69.8
Total	53	100.0

The length of stay at the hospital depends on a large number of factors like the kind of trauma, previous health conditions, age, etc. In our study, the average length of stay was found to be around three to six days while a few patients stayed over a month (Table 3).

**Table 3:** Length of stay (LOS) distribution of patients studied Mean ± SD: 9.62±9.9

LOS	No. of Patients	%
1-2	11	20.8
3-6	19	35.8
7-12	9	17.0
13-18	6	11.3
19-30	5	9.4
>30	3	5.7
Total	53	100.0

Combining the age and gender statistics, Table 4 shows that men less than 30 years seemed to have more fatalities while amongst women, the age criteria did not seem to be different between the various age intervals.

**Table 4:** Age distribution in relation to the gender of patients studied

Age in Years	Gender		Total
	Female	Male	
<30	4(25%)	18(48.6%)	22(41.5%)
30-40	4(25%)	11(29.7%)	15(28.3%)
41-50	4(25%)	3(8.1%)	7(13.2%)
>50	4(25%)	5(13.5%)	9(17%)
Total	16(100%)	37(100%)	53(100%)

Table 5 shows that women had a longer length of stay as compared to men.

**Table 5:** Comparison of age and LOS according to the gender of patients LOS: length of stay p=0.179, Insignificant, Fisher's exact test

Variables	Gender		Total	P-value
	Female	Male		
Age	42.06±17.27	33.21±14.73	35.88±15.91	0.062+
LOS	10.75±13.22	9.13±8.37	9.62±9.97	0.594

Table 6 shows the injuries to various organs among males and females. Injury to the head has been very common in the population while injury to the spleen has been the least common in our study. This proves that wearing a helmet is very important to prevent injury to the head

**Table 6:** Frequency distribution of injuries of patients studied Chi-square test/Fisher's exact test

Variables (Injuries)	Gender		Total (n=53)	P Value
	Female (n=16)	Male (n=37)		
Head	9(56.3%)	24(64.9%)	33(62.3%)	0.553
Liver	3(18.8%)	17(45.9%)	20(37.7%)	0.061+
Spleen	1(6.3%)	1(2.7%)	2(3.8%)	0.534
Renal	2(12.5%)	1(2.7%)	3(5.7%)	0.213

Intra and extra-abdominal injuries are crucial in patients, as they affect the course of treatment, the length of stay, and the amount of bleeding in the patients (Table 7).

**Table 7:** Intra-abdominal/extra-abdominal injuries Chi-square test/Fisher's exact test

Variables (Associated -)	Gender		Total (n=53)	P-value
	Female (n=16)	Male (n=37)		
Intra-abdominal	4(25%)	15(40.5%)	19(35.8%)	0.279
Extra- abdominal	8(50%)	17(45.9%)	25(47.2%)	0.786

From Table 8, it is clear that extra-abdominal injuries are more common.

**Table 8:** Need for extra-abdominal operations

Need for extra-abdominal operations	Gender		Total
	Female	Male	
No	11(68.8%)	27(73%)	38(71.7%)
Yes	5(31.3%)	10(27%)	15(28.3%)
Total	16(100%)	37(100%)	53(100%)

A Grade 3 injury to the liver, spleen, or kidney has the worst prognosis and longer healing time. From our study, liver injuries in both male and female patients were common while renal injuries were not to be found (Table 9).

**Table 9:** Grade of liver injury/splenic injury/renal injury p=0.754, insignificant, chi-square test Chi-square test/Fisher's exact test

Variables	Gender		Total (n=53)	P-value
	Female (n=16)	Male (n=37)		
Grade> 3-liver injury	2(12.5%)	4(10.8%)	6(11.3%)	1.000
Grade> 3-splenic injury	1(6.3%)	0(0%)	1(1.9%)	0.302
Grade> 3-renal injury	0(0%)	0(0%)	0(0%)	1.000

The injury severity score (ISS) is a score to assess the overall severity of injuries in trauma patients. It involves fracture to the mandible, fracture of the lower end of the radius, fracture of the ribs with a flail segment, abrasions, and neck pain. The greater the score, the greater the fatality. See Table 10 for the distribution of patients according to ISS.

**Table 10:** Injury severity score, ISS (75)- Distribution of patients studied p=0.304, insignificant, student's t-test

ISS(75)	Gender		Total
	Female	Male	
1-10	11(68.8%)	20(54.1%)	31(58.5%)
11-20	2(12.5%)	9(24.3%)	11(20.8%)
21-30	2(12.5%)	4(10.8%)	6(11.3%)
31-40	1(6.3%)	2(5.4%)	3(5.7%)
41-50	0(0%)	2(5.4%)	2(3.8%)
Total	16(100%)	37(100%)	53(100%)
Mean ± SD	10.37±10.24	13.72±11.00	12.71±10.79

Body mass index plays a vital role in healing and affects the length of stay at the hospital. Table 11 shows that the males (n=20, 54.1%) more affected had a BMI of 18.5-24.9, whereas females (n=10,62.5%) more affected had a BMI of 25.0-29.9. Table 12 shows the distribution of patients according to body mass index in international values (BMI_INT).

**Table 11:** Body mass index (BMI): distribution of patients studied p=0.300, insignificant, student's t-test

BMI	Gender		Total
	Female	Male	
<18.5	0(0%)	0(0%)	0(0%)
18.5-24.9	6(37.5%)	20(54.1%)	26(49.1%)
25.0-29.9	10(62.5%)	12(32.4%)	22(41.5%)
>30.0	0(0%)	5(13.5%)	5(9.4%)
Total	16(100%)	37(100%)	53(100%)
Mean ± SD	23.87±3.07	24.91±3.43	24.60±3.33

**Table 12:** BMI_INT: distribution of patients studied BMI_INT: body mass index in international values

BMI_INT	Gender		Total
	Female	Male	
Underweight	1(6.3%)	1(2.7%)	2(3.8%)
Normal	5(31.3%)	19(51.4%)	24(45.3%)
Overweight	10(62.5%)	12(32.4%)	22(41.5%)
Obesity	0(0%)	5(13.5%)	5(9.4%)
Total	16(100%)	37(100%)	53(100%)

Table 13 shows that at the time of admission, 56.8% of males underwent a whole-body CT scan when compared to 43.2% of females. Further re-imaging was done in 48.6% and re-visits were considered in 78.4% of patients.

**Table 13:** WBCT/selective (S)/re-imaging/re-visits WBCT: whole-body CT scan p=0.101, insignificant, Fisher's exact test

Variables	Gender		Total	P Value
	Female	Male		
WBCT / S				
S	8(50%)	21(56.8%)	29(54.7%)	0.650
W	8(50%)	16(43.2%)	24(45.3%)	
Re-Imaging				
N	7(43.8%)	18(48.6%)	25(47.2%)	0.743
Y	9(56.3%)	19(51.4%)	28(52.8%)	
Re-visits				
N	9(56.3%)	8(21.6%)	17(32.1%)	0.013
Y	7(43.8%)	29(78.4%)	36(67.9%)	
Total	16(100%)	37(100%)	53(100%)	

Table 14 shows that the majority of patients suffered MVA, with males accounting for the majority (n=25, 67.6%). However, among the female group sustaining injuries, MVAs were the number one cause.

**Table 14:** Mechanism of injury: distribution of patients studied RTA: road traffic accident; BAT: blunt abdominal trauma Chi-square test/Fisher's exact test p=0.238, insignificant, Fisher's exact test

Mechanism of injury	Gender		Total
	Female	Male	
RTA	13(81.3%)	25(67.6%)	38(71.7%)
Assault	1(6.3%)	1(2.7%)	2(3.8%)
BAT	2(12.5%)	2(5.4%)	4(7.5%)
Fall height	0(0%)	6(16.2%)	6(11.3%)
Penetrating injury	0(0%)	3(8.1%)	3(5.7%)
Total	16(100%)	37(100%)	53(100%)

Table 15 showed no relation between comorbidity and length of stay (p=0.419, insignificant).

**Table 15:** Comorbidities: distribution of patients studied DM: diabetes mellitus; HTN: hypertension; MI: myocardial infarction; AF: atrial fibrillation p=0.419, insignificant, Fisher's exact test

Comorbidities	Gender		Total
	Female	Male	
NIL	13(81.3%)	33(89.2%)	46(86.8%)
YES	3(18.8%)	4(10.8%)	7(13.2%)
DM	2(12.5%)	2(5.4%)	4(7.5%)
HTN	0(0%)	1(2.7%)	1(1.9%)
MI	0(0%)	1(2.7%)	1(1.9%)
AF	0(0%)	1(2.7%)	1(1.9%)
Obesity	1(6.3%)	0(0%)	1(1.9%)
Hypothyroidism	1(6.3%)	0(0%)	1(1.9%)
Nutritional deficiency	0(0%)	1(2.7%)	1(1.9%)
Total	16(100%)	37(100%)	53(100%)

Table 16 shows the need for blood transfusion in the first six hours.

**Table 16:** Blood transfusion (BTF) in the first six hours p=0.340, insignificant, Fisher's exact test

BTF in first 6 hrs	Gender		Total
	Female	Male	
No	13(81.3%)	24(66.7%)	37(71.2%)
Yes	3(18.8%)	12(33.3%)	15(28.8%)
Total	16(100%)	36(100%)	52(100%)

The majority of patients had an ISS of ≤10, with 58.3% having normal BMI, 63.6% being overweight, and 40% being overweight. Patients with ISS 41-50 accounted for 3.8%, p=0.796, insignificant, and hence there was no correlation between BMI and ISS. See Table 17.

**Table 17:** Comparison of the injury severity score (ISS) in relation to body mass index (BMI) p=0.185, insignificant, student's t-test, r=0.036; p=0.796

ISS	BMI				Total
	UNDER WEIGHT	NORMAL	OVER WEIGHT	OBESITY	
1-10	1(50%)	14(58.3%)	14(63.6%)	2(40%)	31(58.5%)
11-20	0(0%)	7(29.2%)	4(18.2%)	0(0%)	11(20.8%)
21-30	0(0%)	2(8.3%)	2(9.1%)	2(40%)	6(11.3%)
31-40	0(0%)	0(0%)	2(9.1%)	1(20%)	3(5.7%)
41-50	1(50%)	1(4.2%)	0(0%)	0(0%)	2(3.8%)
Total	2(100%)	24(100%)	22(100%)	5(100%)	53(100%)
Mean ± SD	22.50±26.16	12.12±9.37	10.77±9.98	20.20±13.21	12.71±10.79

## Discussion

Bolandparvaz et al. found that the majority of trauma patients were young adult males [1]. Table 1 shows that the majority of patients in our study were <30 years (n=22, 41.5% with Mean ± SD: 35.88±15.91). Table 2 showed that the bulk of patients (n=37, 69.8%) were males. Individuals with polytrauma ( LOS ≥ 7 days and ISS 12+) had a poorer recovery 12 months post-injury. Hung et al. showed that post-injury mediators have a critical role in determining long-term health consequences [8]. In Table 3, the majority of the patients (n =30, 55.6%) had brief hospitalization (≤5 days, Mean ± SD: 9.62±9.97). However, individuals with higher ISS have a greater length of hospital stay. Tavris et al. showed that hospitalizations in MVAs increased in males [11-13]. Table 4 showed that the majority of patients (n=22, 41.5%) were under the <30 years. Young adults (n=15, 30-40 years old) made up 28.3%, with 25% (n=4) females and 29.7% (n=11) males. Males made up the bulk of the gender distribution (n=29, or 78.3%). Table 5 shows that LOS is higher among females (Age=42.06±17.27 years, LOS= 10.75±13.22 days). Table 6 demonstrates that 62.3% of MVA patients (n=33) had a head injury of varying severity, with the majority being males (n=24).

Thirty-seven point seven percent (37.7%) of patients (n=20) sustained liver injuries of which the majority were males (n=17). Young males (Mean SD: 35.8815.91 years) are at the greatest risk of RTA-related traumatic brain injury (TBI). Table 7 shows that both intra (n=15, 40.5%) and extra (n=17,45.9%) -abdominal injuries were more common in males.

Table 8 shows that men were among the most severely and critically injured; the need for extra-abdominal surgeries (n=37, 100%) was more in this group. Table 9 shows that the majority of patients irrespective of gender sustained Grade > 3 liver injury. A solid-organ injury was more common in men. Table 10 shows the distribution of patients with respect to the injury severity score (ISS). In the age group from one to 10 years, of a total of 31 (58.5%), 54.1% and 68.8% belonged to the male and female groups, respectively. ISS of >75 was common in this age group irrespective of gender. Table 11 shows that males (n=20, 54.1%) more affected had a BMI of 18.5-24.9, whereas females (n=10,62.5%) more affected had a BMI of 25.0-29.9. Here, the Mean +/- SD was 24.60 +/- 3.33. Table 12 shows that obesity and BMI are independent of the outcomes of the trauma irrespective of gender. However, the majority of patients with normal BMI (n=24, 45.3%, p=0.101) are mostly involved in trauma. Table 13 shows that at the time of admission, 56.8% of males underwent a whole-body CT scan when compared to 43.2% of females. Further re-imaging was done in 48.6% and re-visits were considered in 78.4% of patients. Table 14 shows that the majority of patients suffered MVA, with males accounting for the majority (n=25, 67.6%). However, amongst the female group sustaining injuries, MVAs were the number-one cause.

Hwabejire et al. showed that system-related issues, not the severity of illness, prolong hospital stay [9]. Table 15 showed no relation between comorbidity and length of stay (p=0.419, insignificant). Table 16 showed the need for blood transfusion in the first six hrs after trauma. Fifteen patients (28.8%) needed a transfusion. The majority of them were males (n=24, 66.7%).

Although massive blood transfusion can be linked with independent risk variables, Velmahos et al. concluded that discontinuing short-term treatment cannot be justified based on the necessity for massive blood transfusion [11]. Table 17 shows a comparison of the injury severity score (ISS) in relation to BMI. The majority of patients had an ISS of ≤10, with 58.3% having normal BMI, 63.6% being overweight, and 40% being overweight. Patients with ISS 41-50 accounted for 3.8%, p=0.796, insignificant, and hence there was no correlation between BMI and ISS [14].

## Conclusions

The bulk of the patients in our three-cycle audit were young male adults <30 years. Females have a higher LOS than males. Prolonged hospitalization is associated with significant clinical risks and increased costs. Individuals with severe injuries (those with LOS ≥ 7 days and ISS 12+) had poorer recovery 12 months after the injury. The majority of patients (n =30, 55.6%) had brief hospitalization (≤5 days). Men sustained the majority of the head injuries and were the worst affected, necessitating extra-abdominal surgery. The majority of patients sustained a Grade > 3 liver injury. An ISS of >75 was common in the age group of 1-10 years. Our research found no link between comorbidities and length of stay in MVA patients. Though the majority of patients did not require surgery, 28.8% required a blood transfusion. Our research found no link between BMI and ISS.

Obesity and BMI are not associated with trauma outcomes. Obese people sustaining MVAs had the same injured body regions as patients who were normal weight. Although they had a lower ISS than normal-weight individuals, they had a longer in-hospital LOS. The last cycle of our audit showed that people who had their safety gear and helmet on had a smaller length of stay and low severity of injuries.
